# Maternal folate receptor alpha autoantibodies and folate levels during pregnancy in relation to autism spectrum disorder: a pilot case-control study

**DOI:** 10.3389/fpsyt.2026.1914371

**Published:** 2026-09-23

**Authors:** Olga Egorova, Erik Domellöf, Sven Arne Silfverdal

**Affiliations:** 1 Department of Clinical Sciences, Pediatrics, Umeå University, Umeå, Sweden; 2 Department of Cognitive Medicine, Ängelholm Hospital, Ängelholm, Sweden; 3 Department of Psychology, Umeå University, Umeå, Sweden

**Keywords:** autism spectrum disorder, folate, folate receptor alpha autoantibodies, neurodevelopment, pregnancy

## Abstract

**Background:**

Folate receptor alpha autoantibodies (FRAA) are reported to be associated with autism spectrum disorder (ASD) and are found more frequently in mothers of children with ASD. However, the relationship between maternal FRAA during pregnancy, folate status, and ASD risk in offspring remains unclear.

**Objective:**

In this exploratory pilot study, we compared maternal serum binding FRAA measured during pregnancy between high-folate ASD cases and childbirth-time–matched mothers of typically developing children.

**Methods:**

FRAA were retrospectively analyzed in serum samples collected between gestational weeks 7 and 18 from women whose children were later diagnosed with ASD (case subgroup, n = 10) and mothers of neurotypical children (control subgroup, n = 10). Case samples were selected based on previously measured elevated folate levels, while controls were selected to minimize differences in biobank storage duration.

**Results:**

FRAA were detected in 2 of 10 samples in each group, corresponding to a prevalence of 20%. Although based on limited statistical power, no significant difference in FRAA prevalence was observed.

**Conclusion:**

In this exploratory pilot study, no difference in binding FRAA prevalence was found between mothers of children with ASD, who were selected for elevated maternal folate levels, and mothers of neurotypical children. While this finding does not support a link between FRAA and elevated maternal folate levels, the small sample size and selection criteria limit interpretation.

**Limitations:**

The study was limited by the small sample size, differences in subgroup selection, uncertainty in gestational age at sampling, and the inability to assess blocking FRAA.

## Background

Autism Spectrum Disorder (ASD) is an umbrella term for a highly heterogeneous group of neurodevelopmental conditions (1). Research over the last decade indicates the importance of autoantibodies against folate receptor alpha (FRAA) in the ASD etiology (2–4), as well as elevated prevalence of FRAA in mothers of children with ASD compared to mothers of typically developing children (5, 6). Especially harmful are FRAA blocking interaction between folate receptor alpha and folate (3, 7). However, maternal autoimmunity in these studies was evaluated years postpartum. Consequently, whether FRAA were present during pregnancy and influenced fetal development remains undetermined.

Folate receptor alpha (FRα) serves as the primary high-affinity folate transporter across the placenta (8), and its activation is critical throughout the development of the nervous system (9). Mechanistically, FRα may serve as a transporter of maternal FRAA through the blood-placental barrier into fetal circulation. Once in fetal circulation, FRAA may potentially affect fetal CNS development, preventing folate transport (6).

Maternal folate supplementation was often pointed to as a potential factor lowering liability for ASD in offspring (10). However, while maternal folate status has a strong correlation regarding offspring neural tube defects, results are inconsistent in the case of ASD (11). While studies of maternal folate supplementation have generally reported positive effects, studies assessing maternal blood folate levels yielded nonlinear associations (12–15), which may reflect a complicated blood-to-cell folate transport system (9).

In a previous retrospective study, we observed an unexpected positive correlation between maternal serum folate levels and the risk of an ASD diagnosis in children (16). Theoretically, elevated folate levels may result from good supplementation and/or decreased blood-to-cell folate transport (17), for example, due to FRAA-FRα interaction. The objective of this study was to explore whether comparatively high serum folate concentrations during pregnancy, observed in mothers pregnant with a child later diagnosed with ASD, may be explained by inhibition of folate transport via folate receptor-α caused FRAA. This hypothesis was supported by the observation that a greater proportion of serum samples from mothers of children with ASD contained unmetabolized folic acid compared with samples from control mothers (16). Folic acid is transported into cells primarily via FRα (18). Thus, FRAA may interfere with folic acid transport and its conversion into physiologically active folate. In this pilot study, serum samples with predefined folate levels were used (16). Samples from mothers of children with ASD were selected on the basis of high folate concentrations and compared with serum samples from mothers of typically developing children matched by childbirth timing to ensure comparable biobank storage duration.

Methodically, it is possible to differentiate the FRAA-subtype that blocks interaction between folate and FRα (3), which results in decreased folate transport across biological barriers (19). In the present exploratory pilot study, however, biobank restrictions allowed only the binding FRAA assay to be performed. This assay detects all autoantibodies that bind to folate receptor alpha regardless of their blocking activity (3). Such antibodies have been reported in up to 30% of individuals in some populations (7, 20).

## Methods

This retrospective pilot study was conducted in a subgroup of the groups described in a previous publication (16).

*Previous study cohort:* Women residing in Västerbotten County during pregnancy and until their child either received an ASD diagnosis (the case *group*) or, for the control *group*, reached at least 5 years of age. The inclusion procedure has been described previously (16). Mothers of children born between 1996 and 2009 were eligible (**Table 1**).

**Table 1:** Baseline characteristics, complete group n=200 16 and subgroup, analyzed for FRAA n=20.

Maternal and child characteristics	Case		Control		Case-Control differences^a^
	n	Median (IQR) or %	n	Median (IQRa) or %	p
Complete group (n=200)					
Mother’s age (years)	100	30(26-33)	100	32(28-35)	0.02
Year of blood serum sampling	100	2002(1999-2005)		2003(2001-2005)	0.08
Month of birth, child	100	8(4-10)	100	7(4-10)	0.47
Child sex, boys	78	78%	76	76%	0.87
ASD diagnosis					
Infantile autism (F84.0)	74	74%			
Asperger’s syndrome (F84.5)	26	26%			
Subgroups, analyzed for FRAA (n=20)					
Mother’s age (years)	10	32(26 – 36)	10	32.5(32 – 35)	0.76
Year of blood serum sampling	10	2004(2001-2005)	10	2004(2001-2005)	0.94
Month of birth, child	10	8 (4 -10)	10	7 (5 – 10)	0.97
Child sex, boy	8	80%	8	80%	1
ASD diagnosis					
Infantile autism (F84.0)	7	70%			
Asperger’s syndrome (F84.5)	3	30%			

^a^ Test for difference with Mann-Whitney U test (continuous variables) or Fisher-exact test (categorical variables).

ASD diagnoses (infantile autism, F84.0, or Asperger syndrome, F84.5) had been made within the health care system according to DSM-IV (21), upon referral by parents or caregivers and before the start of this study, ensuring that assessors were blinded to maternal biomarker data. Exact age of diagnosis was not available, although infantile autism in Sweden is typically diagnosed at 4–5 years of age and Asperger syndrome after age 7.

*Sample collection and biobanking*: Serum samples were routinely collected for rubella testing during the first midwife visit. The time point of the first visit is at the discretion of the patients and occurs in most cases between weeks 7 and 18, centering around week 14 (22, 23). As we did not have access to the exact gestational age at blood sampling, we assigned all patients to week 14 (23). For women who consented, residual serum was stored in the Northern Sweden Maternity Cohort biobank and made available for research purposes (22, 23).

*Selection criteria for the present pilot study:* Serum samples had previously been analyzed for 64 metabolites, including folate species such as 5-methyl-tetrahydrofolate (mTHF) and 4α-hydroxy-5-methyl-tetrahydrofolate (hmTHF) (16). Total folate was defined as mTHF + hmTHF.

From the twenty ASD-group samples with the highest total folate levels, ten samples available under biobank rules were included (case *subgroup*). Control samples (control *subgroup*) were selected using a frequency-balancing approach to match the storage duration of the case samples, because FRAA titers may decrease during prolonged storage (3) (**Figure 1**; **Table 1**). Consequently, control samples did not necessarily represent the highest folate levels within the initial control group (**Figure 2**; **Table 2**). In the case mother-child pair subgroup used in the present study, seven children met ICD-10 criteria for infantile autism and three for Asperger syndrome (**Table 1**).

**Figure 1:**
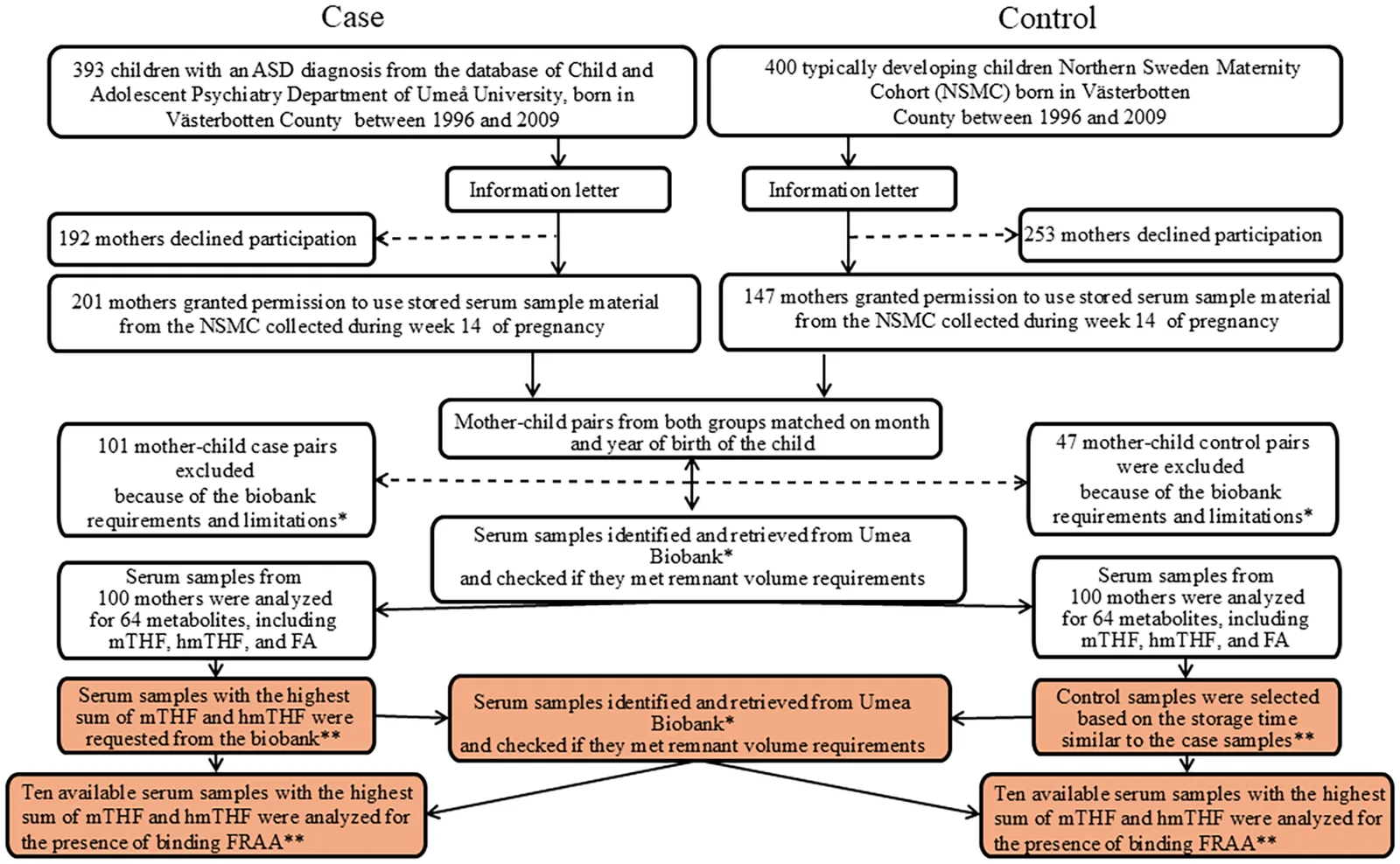
Study design. Details with a *white background* refer to the inclusion procedure for folate testing and were previously published (16). Children were born between 1996 and 2009. All ASD diagnoses had been established by the health care system according to DSM-IV previous to the project start. In the mother-child case subgroup for the present study, 7 children met the ICD-10 criteria for infantile autism (F84.0) and 3 the criteria for Asperger syndrome (F84.5). Details with a *grey background* show inclusion for FRAA testing for the current pilot study. * The sample volume was restricted to 200 µL by the biobank steering committee, and only samples with a remnant volume greater than 700 µL were eligible for our study. ** Eight serum samples included in the case subgroup belong to the samples with the top ten highest folate levels in the case group. *** Due to different inclusion criteria, folate levels in the control subgroup were not the highest measured in the entire control group; only two samples belong to the samples with the top ten highest folate levels in the control group. Adapted with permission from Maternal blood folate status during early pregnancy and occurrence of autism spectrum disorder in offspring: a study of 62 serum biomarkers by Egorova et al., Molecular Autism, 2020 (16).

**Figure 2:**
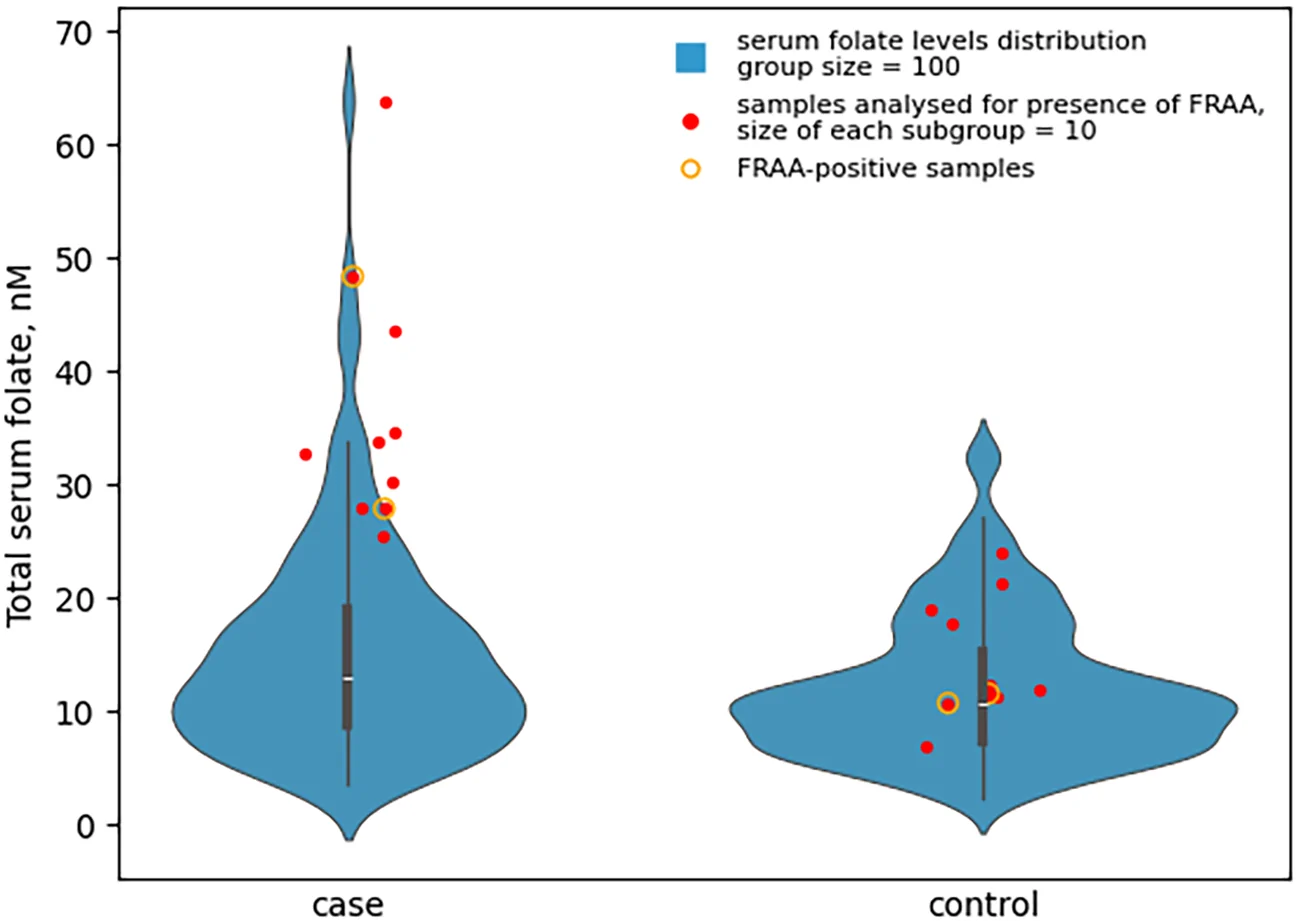
Maternal serum folate distributions in case and control groups, and FRAA-positive sample markers in case and control subgroups. *Blue violin plots* show the distribution of serum folate levels in the previously analyzed cohort. The case group includes serum samples collected between the 7th and 18th gestational week from mothers of children later diagnosed with ASD, and the control group includes samples collected during the same gestational period from mothers of typically developing children. Each group consisted of 100 participants. *Red dots* indicate serum folate levels in the subset of samples analyzed for the presence of binding FRAA (10 samples per group). FRAA-positive samples are highlighted with *orange circles*. Serum folate concentrations are reported in nmol/L (nM).

**Table 2:** Folate levels in the entire recruited population and in the subgroup.

Metabolites	Median (IQR, min-max), nM/L or N(%)			Shapiro–Wilk normality test (p-value)		Case-Control differences^a^
	Case	Control		Case	Control	p
Complete cohort (n=200)						
Total folate (sum of mTHF and hmTHF) (n=100)	12.90 (8.91– 19.02, 3.58 – 63.90)		10.74 (7.29 – 15.26, 2.33 – 32.80)	< 0.001	< 0.001	0.01
Subgroups, analyzed for FRAA (n=20)						
Total folate (sum of mTHF and hmTHF) (n=10)	33.29 (28.49 – 41.43, 25.58 – 63.90)		12.20 (11.46–18.65, 6.90 – 24.12)	0.357	0.043	< 0.001
FRAA prevalence	2(20%)		2(20%)	–	–	1
unmetabolized folic acid	7(70%)		4(40%)	–	–	0.37

^a^ Test for difference with Mann-Whitney U test (continuous variables) or Fisher-exact test (categorical variables).

Although the differing sampling criteria introduce an inherent selection bias, this strategy was chosen to maximize the likelihood of detecting any difference in FRAA prevalence within the constraints of a limited sample.

*FRAA assay*: Twenty pseudonymized serum samples were shipped frozen on dry ice via secure transport to Religen (USA) for analysis. Samples had been stored at -80 °C until shipment and underwent three freeze–thaw cycles before analysis. All laboratory personnel were blinded to participants’ clinical status and sample origin.

Folate receptor-α autoantibodies (FRAA) were measured using the ELISA-based binding assay described in (3), which is routinely performed at Religen. The assay quantifies autoantibodies that bind to folate receptor-α, accessing both FRAA that only bind to the folate receptor-α (FRα) as well as FRAA that block FRα-folate interaction. Results were reported as optical density (OD) units, and according to information provided by Religen, samples were considered positive when the OD value exceeded 0.250. Assay precision metrics (intra- and inter-assay CVs) for the blocking FRAT assay have not been published.

*Statistics*: Statistical analyses were conducted using IBM SPSS Statistics (version 30.0.0.0) and verified using the *scipy.stats* module in Python (scipy version 1.11.4). Because of the small sample size (n = 10 pairs) and violation of normality assumptions, intergroup differences were evaluated using the non-parametric Mann–Whitney U test and exact distribution method. FRAA proportions were compared with Fisher’s exact test. Continuous variables are reported as median (interquartile range = IQR). All statistical tests were two-sided, and p < 0.05 was considered statistically significant.

*Ethics.* All participants received written information about the study, and only those who provided written informed consent were included (16).

## Results

### Demographics

Age at sampling, month of childbirth, and child gender did not differ between case and control subgroups. The previously reported age difference (30.0 vs. 32.0 years, p = 0.02) refers to the *initial cohort* of 100 cases and 100 controls. In contrast, in the present pilot FRAA subgroup (10 vs. 10), maternal age did not differ significantly (32.0 vs. 32.5 years, p = 0.76) (**Table 1**).

### Folate levels

Total serum folate levels differed between subgroups (**Table 2**). Median [IQR] folate levels were higher in the case subgroup (33.29 nM [28.37–45.39]) than in the control subgroup (10.74 nM [11.46–18.65], p < 0.001). In the full cohort (n = 100 per group), the difference was smaller (12.90 [8.91–19.02] vs. 12.20 [7.29–15.26] nM, p = 0.01). Folate concentrations in almost all samples were within the expected physiological reference interval, even if serum folate levels in the case subgroup were within the upper part of the expected reference interval for the second trimester (24).

In the case subgroup, serum folate levels were higher, ranging from 25.28 nM to 63.90 nM, and seven of ten samples (70%) were positive for unmetabolized folic acid (**Table 2**). In the control subgroup, folate levels ranged from 6.90 nM to 24.12 nM; four samples (40%) in this subgroup were positive for unmetabolized folic acid. A Fisher’s exact test indicated no statistically robust difference in the prevalence of unmetabolized folic acid between the subgroups (p = 0.39, **Table 2**).

The larger difference in folate concentrations observed in the subgroups selected for the present study (**Figure 1**, grey part) has been produced by intentional selection of samples with the highest folate levels into the case subgroup and skipping this criterion in the control subgroup. The observed folate distributions confirm that the ASD subgroup selected for FRAA analysis represented mothers with particularly elevated folate concentrations, consistent with the study objective of exploring whether elevated maternal folate levels were associated with increased FRAA prevalence.

### FRAA

The ELISA-based binding assay was positive in 2 of 10 samples in both the case and control subgroups, corresponding to a prevalence of 20% (95% CI: 2.5%–55.6%) in each subgroup, indicating that FRAA was present in these samples. Comparison of FRAA-positivity between groups (2/10 vs. 2/10) using Fisher’s exact test (two-sided) showed no difference, as expected for the present distribution (p = 1.0; **Table 2**).

Two of the FRAA-positive samples in the case subgroup belonged to mothers of children later diagnosed with Asperger syndrome (DSM-IV code F84.5) and infantile autism (DSM-IV code F84.0), with total serum folate levels of 48.4 nM and 27.9 nM, respectively. Only the sample belonging to the mother of the child with diagnosis code F84.5 contained unmetabolized folic acid (folic-acid-positive). In the control subgroup, total serum folate levels in the two FRAA-positive samples were 11.59 nM and 10.75 nM (**Figure 2**); none of these two samples were folic-acid-positive.

## Discussion

The present exploratory pilot study evaluated FRAA in maternal serum samples with available folate concentration data, nested within a larger previously analyzed sample set. The objective was to examine whether the high serum folate concentrations observed in a subset of mothers of children with ASD could be explained by inhibition of folate transport via folate receptor-α (FRα) caused by autoantibodies to the receptor (FRAA). This sampling strategy was chosen to minimize potential bias in FRAA positivity related to storage duration, while acknowledging that it introduces its own selection bias. FRAA measurements were performed in samples collected during pregnancy, reducing potential bias from postnatal changes in immune status. Additional strengths include ASD diagnosis of children made independently of the study, blind laboratory processing, and use of a standardized diagnostic assay in a single specialized laboratory.

FRAA-positivity was observed in an equal proportion (20%) of serum samples from mothers of children who were later diagnosed with ASD and from mothers of typically developing children. At face value, this result does not support the hypothesis that elevated folate levels in mothers of children with ASD may be explained by FRAA, as only two samples with high serum folate were FRAA-positive. However, the confidence interval within this sample size (between 2.5% and 55.6%) does not allow us to infer equal FRAA prevalence between the two groups. Notably, the upper limit of this interval is consistent with the higher FRAA prevalence previously reported in mothers of children with ASD (5).

It is noteworthy that FRAA-positive samples in the control subgroup had folate concentrations close to the median of the entire control group and therefore did not exhibit particularly high folate levels (**Figure 2**; **Table 2**). Additionally, among samples analyzed for FRAA, seven of ten samples in the case subgroup and four of ten in the control subgroup contained unmetabolized folic acid. Sweden has no folic acid fortification program (25); thus, folic acid in serum indicates usage of folate supplementation. Even if the differences are not statistically robust, this pattern suggests that more women in the case subgroup with high serum folate used folic acid supplementation and/or used higher doses, which would partially explain the elevated serum folate concentration in these samples (26).

All folic-acid–positive samples in the control subgroup were FRAA-negative, whereas one of the seven folic-acid–positive samples in the case subgroup was FRAA-positive. FRα has higher affinity for folic acid than for metabolically active folates (18), while the other major folate transporter, reduced folate carrier, has extremely low affinity to folic acid (27, 28). Therefore, folic acid supplementation may not alleviate cellular folate deficiency caused by blocking FRAA (3, 7, 29). Assuming this folic-acid-positive sample contained blocking FRAA, both the presence of FRAA and use of folic acid supplementation may have contributed to the elevated serum folate levels for this woman. However, the FRAA positivity in this study was defined solely by the binding assay, which detects all FRAA regardless of their ability to block the FRα-folate interaction (3). Consequently, neither presence nor absence of blocking FRAA in FRAA-positive samples can be determined. Blocking FRAAs are known to impair CNS development (5, 30), have been associated with reduced fertility (31) and increased risk of neural tube defects (32). Our result does not exclude the possibility that FRAAs in samples from the case subgroup may be of the blocking type (3).

Autoantibody measurements may be influenced by gestational age, as pregnancy is associated with physiological changes in immunoglobulin concentrations and maternal immune regulation (33–35). FRAA titers are also known to fluctuate over time and may decrease below the assay’s detection threshold (31). Therefore, the uncertainty in the timing of sample collection and the reliance on a single measurement should be considered when interpreting FRAA results. Because of the retrospective design, FRAA could not be assessed at multiple time points during pregnancy, and additional participants may have been FRAA-positive at other stages of gestation. However, the pattern observed in this pilot sample does not support our initial assumption that high serum folate concentrations measured at the beginning of the second gestational trimester in selected study samples can be primarily explained by FRAA positivity. Thus, our results cannot be considered supportive of a direct association between serum folate levels and FRAA positivity, even if they neither exclude nor confirm a possible association between maternal FRAA positivity and child diagnosis.

An elevated prevalence of FRAA was reported in mothers of children with ASD than in control populations, as well as in children with ASD, their fathers, and their typically developing siblings (5). In that study, maternal FRAA was measured only after the child had already been diagnosed with ASD, leaving unresolved whether maternal FRAA is associated with the child’s developmental disorder or whether the elevated prevalence observed in ASD-affected families reflects another shared underlying factor. More recent publications, found a stronger association between paternal and children’s FRAA-positivity than between maternal and child’s FRAA-positivity (6), and report that FRAA positivity develops more frequently in individuals carrying the more efficient variant of the reduced folate carrier gene (36). However, comparison with the present study is limited by differences in population selection, timing of sample collection, and assay methodology. Given the limited sample size and exploratory design, the results should be regarded only as hypothesis-generating. A larger sample with unselected participants, repeated measurements across pregnancy, and inclusion of a blocking FRAA assay will be required to clarify the relationship between maternal FRAA status, serum folate levels, and offspring risk for ASD.

### Limitations

This study has several important limitations.

First, each subgroup included only ten participants, resulting in very low statistical power. Assuming FRAA prevalences of approximately 60% in mothers of children with ASD and 20% in control mothers, as reported previously, the estimated power was only about 30%. Therefore, the absence of an observed difference should not be interpreted as evidence of equivalence, and clinically meaningful differences between subgroups cannot be excluded.

Second, ASD cases were selected based on high maternal folate concentrations, whereas controls were not, introducing a selection difference that limits comparability between groups. Although this approach was intentionally chosen to analyze the samples contributing to the previously observed folate differences between mothers of children with ASD and mothers of typically developing children, it does not allow conclusions about any relationship between folate levels and FRAA positivity.

Third, FRAA were assessed at a single time point during pregnancy, and the exact gestational age at sampling was unavailable. As a result, potential differences in sampling timing and temporal variation in antibody levels could not be evaluated.

Fourth, the number of samples available for FRAA analysis was constrained by biobank regulations, sample availability, and limited serum volume. Consequently, only binding FRAA could be measured, while blocking FRAA could not be assessed due to insufficient remaining material. This limitation prevents evaluation of the relative contribution of different FRAA subtypes, and a potential role of blocking FRAA in maternal folate metabolism or ASD risk cannot be excluded.

## Conclusion

This exploratory pilot study found no observable difference in FRAA prevalence between mothers of children with ASD selected for elevated serum folate concentrations and control mothers. However, the small sample size, retrospective design, subgroup selection criteria, and the inability to assess blocking FRAA limit interpretation and do not allow meaningful differences to be ruled out. Larger studies with unselected participants, precise gestational age information, detailed data on vitamin supplementation, and repeated measurements across pregnancy are needed to clarify the relationship between maternal FRAA status, folate metabolism, and neurodevelopmental outcomes.

## Data Availability

The raw data supporting the conclusions of this article will be made available by the authors, without undue reservation.
